# Thoracic CT Angiographies in Children Using Automated Power Injection with Bolus Tracking Versus Manual Contrast Injection: Analysis of Contrast Enhancement, Image Quality and Radiation Exposure

**DOI:** 10.3390/diagnostics15091103

**Published:** 2025-04-26

**Authors:** Jochen Pfeifer, Deborah Driulini, Katrin Altmeyer, Gudrun Wagenpfeil, Martin Poryo, Christian Giebels, Arno Bücker, Alexander Massmann, Hashim Abdul-Khaliq, Peter Fries

**Affiliations:** 1 Department of Pediatric Cardiology, Saarland University Medical Center, 66421 Homburg, Germany; 2 Clinic for Diagnostic and Interventional Radiology, Saarland University Medical Center, 66421 Homburg, Germanypeter.fries@uks.eu (P.F.); 3 Institute of Medical Biometry, Epidemiology and Medical Informatics, Saarland University Medical Center, 66421 Homburg, Germany; 4 Department of Cardiovascular Surgery, University Heart Center Freiburg-Bad Krozingen, 79189 Bad Krozingen, Germany; 5 Department of Radiology and Nuclear Imaging, Robert Bosch Hospital, 70376 Stuttgart, Germany

**Keywords:** computed tomography angiography, contrast medium, image quality, manual injection, automated power injector, bolus tracking, congenital heart disease

## Abstract

**Objectives:** The purpose of this study was to analyze image quality and radiation exposure of thoracic computed tomography angiography (CTA) in children with congenital heart diseases (CHDs) using either manual contrast medium (CM) injection or automated power injectors with bolus tracking. **Methods:** A total of 137 thoracic CTAs of 120 consecutive pediatric patients were included in this retrospective study. We analyzed the method of CM administration (power injection with bolus tracking (PI) or manual injection (MI)), injection routes, volumes and flow rates of CM. For the evaluation of objective image quality, attenuation values in the heart chambers and great thoracic vessels were determined by region-of-interest (ROI) analysis and signal-to-noise (SNR) and contrast-to-noise (CNR) ratios calculated thereof. Visual image quality was assessed by two blinded readers (four-point Likert-scale) analyzing the presence of artifacts and the depiction of relevant anatomical structures. Effective radiation doses were calculated with dose length products and specific conversion factors. **Results:** CM administration was performed using PI in 119/137 CTAs, whereas MI was conducted in 18/137. The smallest size of peripheral venous cannulas was 24 gauge in 36/137 (26.3%) cases. Overall mean CM volume was 17 mL ± 16 mL (mean ± SD). In PI, the mean flow rate of CM was 1.52 ± 0.90 mL/s with a range between 0.5 and 5.0 mL/s. When comparing the overall PI population and an age-, size- and weight-matched PI subpopulation (18 cases) with the MI population, attenuation values in Hounsfield units (HU) and CNR values were significantly higher in the PI groups than in the MI group for each relevant cardiac structure (left ventricle, right ventricle, ascending aorta and pulmonary trunk, *p* = 0.02–0.001). Overall image quality and depiction of cardiac structures were rated significantly better in CTAs with PI (interquartile ranges: “good” to “excellent” (Likert 3–4)) in PI compared with CTAs acquired with MI (interquartile ranges: “fair” to “good” (2–3)) in MI by both readers (*p* < 0.001). The inter-observer reliability was strong, with a Kendall’s Tau-b correlation coefficient of *τ* = 0.802 (*p* < 0.001). The mean effective radiation dose (E) did not differ significantly when comparing the stratified samples (i.e., the matched PI subgroup and the MI group; 0.5 (±0.3) mSv in both, *p* = 0.76). There were no complications associated with the CM injections for both application approaches. **Conclusions:** Automated contrast agent applications with power injectors and bolus tracking ensure better image quality in pediatric CTA, even when low volumes and flow rates need to be applied. There is a slight increase in radiation associated with bolus tracking. This approach represents a suitable imaging technique for the work-up of congenital heart disease.

## 1. Introduction

Congenital and acquired heart defects in children may present with complex anatomical anomalies and morphological disorders. Imaging techniques are therefore crucial for initial diagnosis as well as for follow-up examinations and surgical planning [1,2,3,4,5].

In most instances, transthoracic echocardiography is the first choice, for example, in case of cardiac murmurs, cyanosis or clinical symptoms of cardiac failure. It provides high-resolution images of cardiac anatomy and morphology. Further clinical indications include the evaluation of cardiac function as well as the hemodynamics, heart valves and shunts in real time [6,7,8]. However, it is limited by inter-observer variability and anatomical restrictions such as small acoustic windows. For further diagnostics, invasive cardiac catheterization and angiography are considered, which allow assessment of hemodynamics and pressure values as well as visualization of the cardiovascular anatomy. However, these invasive procedures may be associated with vascular, thrombotic or embolic complications [9,10,11].

In recent years, computed tomography angiography (CTA) of the cardiovascular system has undergone significant technical improvements. In particular, the introduction of scans with low tube voltage and automatic tube current modulation, high-pitch images in combination with ECG synchronization and iterative reconstruction algorithms have significantly reduced the radiation exposure during these examinations. This has also brought this technique into focus for pediatric patients with heart diseases [12,13,14]. It offers detailed information on cardiac and vascular anatomy, extracardiac tissues and organs as well as their spatial arrangement in relation to each other [15,16,17,18,19]. In particular, three-dimensional reconstruction of CTA datasets contributes to the planning of surgical and interventional procedures. Compared to magnetic resonance angiography, CTA provides a higher spatial resolution and considerably shorter acquisition time so that significantly fewer sedatives need to be administered if necessary.

However, due to the small body size and small vessels in children, safe application of contrast media (CM) is challenging. Especially in neonates and infants, CM has to be injected via small venous lines. CM volumes and flow rates are low and not comparable to those applied in adult patients, in particular when scanning the cardiovascular system [20].

Whether performing hand injection [21] or using power injectors (PIs), CM application has to be conducted with caution with special regard to the volumes and flow rates applied [22]. The main advantage of automated contrast agent injection is that the contrast agent flows reproducibly and homogeneously into the target vessel or organ, which leads to uniform enhancement [23,24]. In contrast, manual injection (MI) can be influenced by the high viscosity of the agent and the force required to generate sufficient pressure on the syringe. This can lead to a heterogeneous flow of the contrast agent in the target vessel and inconsistent image quality. Moreover, the short circulation time in young children may force the examiner to remain in the scanner room after the bolus injection, as the CT scan must be started during or shortly after bolus administration. This can lead to additional radiation exposure for the staff.

In addition to the mode of contrast agent application itself, the timing of the scan acquisition plays an important role for the image quality of the CTA study. In general, start of CT acquisition can be determined on the basis of personal experience or empirical data. However, in patients with altered hemodynamics such as congenital heart disease, this approach can lead to the scan not being acquired at the bolus peak [25,26]. Test bolus techniques and bolus tracking are well-established approaches, especially for cardiovascular imaging, but result in additional radiation respectively contrast agent doses [26,27,28].

The main disadvantage of CTA is the radiation exposure associated with the potential risk of tissue damage or the development of cancer [29,30]. It is generally recognized that radiation doses for medical examinations should be as low as reasonably achievable while providing studies with sufficiently high image quality for diagnostic purposes [31,32].

Previous studies analyzed different scan modes and the radiation exposure using modern dual-source CT (DSCT) in children with congenital heart disease (CHD) [33,34]. However, there were only a few studies published particularly analyzing the image quality of CTA in children in regard to the CM application mode.

In our study, we analyzed image quality, radiation exposure and acute complications of contrast-enhanced CTA studies in children acquired with a third-generation dual-source scanner using either MI or automated PI with bolus tracking for contrast agent application. The aim was (1) to assess CM access routes, volumes and flow rates as well as CM-associated complications and (2) to evaluate whether there is a difference in image quality and radiation exposure depending on the method of CM administration in pediatric CTA.

## 2. Materials and Methods

This single-center retrospective cohort study was approved by the local ethics committee (Ärztekammer des Saarlandes, Saarbrücken, Germany; file number 93/21). It was performed at a tertiary care medical center (University Medical Center, Homburg, Germany).

### 2.1. Study Population

We retrospectively analyzed all children with cardiovascular diseases who underwent thoracic CTA with ECG synchronization from January 2016 to January 2021. Inclusion criteria were CTA with contrast injection either by hand or by automated power injector with bolus tracking, diagnosis of congenital or acquired cardiovascular disease and age under 18 years. Exclusion criteria included age over 18 years, CTA studies performed without ECG synchronization or CT studies of the thorax for clinical indications other than evaluating the cardiovascular system.

The parents had given written informed consent before the CT examination. Based on the retrospective nature of this data analysis, detailed written informed consent of the patients and their chaperones for the conduction of this research study was waived. We reviewed the relevant patients’ files and CTA records in order to obtain the following data:

- Patients’ characteristics (sex, age, body weight, body mass index at the time of CTA),
- CM volumes and flow rates,
- Venous access routes and sizes of intravenous lines for CM application,
- Method of CM administration (MI or PI),
- Scan parameters (tube voltage, volume computed tomography dose index (CTDI_vol_), dose length product (DLP), calculation of effective doses (E)),
- CT attenuation values for the calculation of signal-to-noise ratios (SNRs) and contrast-to-noise ratios (CNRs),
- Complications associated with CM administration (extravasation, air embolism and allergic reaction).

All data obtained were anonymized at the source before further processing and evaluation.

### 2.2. Image Acquisition and Image Processing

All CTA studies enrolled were performed with ECG synchronization using a third-generation dual-source CT scanner (SOMATOM© Force, Siemens Healthineers, Erlangen, Germany) with automated radiation exposure control (CAREkV and CAREdose4D). The CTA was performed using a high pitch acquisition (pitch: 3.2) with a collimation of 2 × 192 × 0.6 mm. Baseline tube voltage was set to 70 kVp and baseline tube current set to 100 mAs. Standard image reconstructions included 0.6 mm axial images in soft tissue (Br40) and lung tissue (Bl64) kernels. In addition, 1 mm thick multiplanar reconstructions in axial, coronal and sagittal orientations were generated from raw data.

Highly concentrated iodinated contrast agent (Imeron© (Iomeprol 400), Bracco Imaging SpA, Milan, Italy) was administered either by hand injection or using an automated dual-head power injector (Accutron CT-D, Medtron AG, Saarbrücken, Germany) at a dose of 1 mL per kilogram of body weight followed by an injection of 10–20 mL of sterile saline solution. The decision of whether to perform MI of the contrast agent or to use PI and the underlying flow rates for the automated injection was made based on the clinical situation and on discretion of the attending radiologist and pediatric cardiologist.

To ensure optimal timing of the CT scan for the arrival of the CM in the target vessel, bolus tracking (with CARE Bolus) was performed in the PI group. With this tool, we acquired one axial image/sec (tube voltage: 70 kV, tube current: 10 mAs) at the level of the heart with the tracking region of interest (ROI) being placed within the left ventricle. CTA scan acquisition was automatically started at a predefined trigger threshold of 80 Hounsfield units (HU).

In case of MI, CT scan acquisition was started after termination of the CM and saline chaser bolus injection without the use of bolus tracking.

Anti-tachycardia medication, such as beta-blockers, had not been applied before the CTA. All examinations were supervised by a radiologist and a pediatric cardiologist. Possible complications in terms of extravasation of contrast medium and allergic reaction were recorded.

### 2.3. Analysis of Objective Image Quality

**Figure 1:**
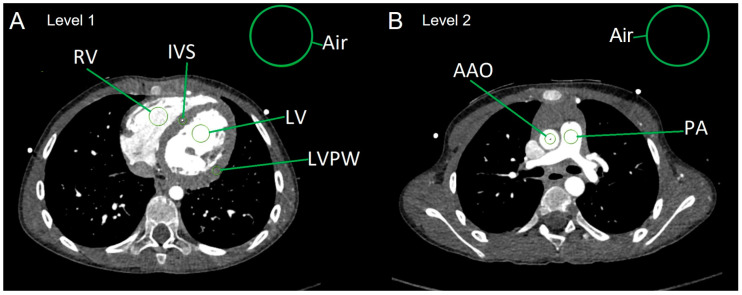
Measurement of CT attenuation values with regions-of-interest (**A**) at level 1 (IVS: interventricular septum; LV: left ventricle; LVPW: LV posterior wall; RV: right ventricle) and (**B**) at level 2 (AAO: ascending aorta; PA: main pulmonary artery). Standard deviation of the attenuation values obtained from the ROI placed in the air outside of the body served as background noise.

All CTA studies included in this study were anonymized for further evaluation. CT attenuation values measured in HU were obtained from ROIs placed in relevant anatomical structures at two levels on axial 0.6 mm soft tissue reconstructions using a conventional Picture Archiving and Communication System (PACS) work station (Sectra IDS7, Version 24.2, Sectra AB, Linköping, Sweden) (Figure 1).

Level 1 was located at the level of the heart displaying the ventricles in the axial plane. ROIs were placed at the cavum of the left ventricle (LV) and of the right ventricle (RV), the myocardium of the interventricular septum (IVS) and the left ventricular posterior wall (LVPW), respectively, with the Hounsfield units (HU) obtained thereof. The HU standard deviation (SD) of a ROI placed in the air outside the body at this level was also recorded and considered as image noise [35,36].

Level 2 was the axial plane at the level of the pulmonary bifurcation. Here, ROIs were placed at the ascending aorta (AAO) and the main pulmonary artery (PA). Again, the HU standard deviation (SD) of the air was considered as image noise.

Average myocardial HUs were calculated in the following way:
HU[myocardium] = (HU[IVS] + HU[LVPW])/2.
Calculation of image parameters SNR and CNR:

The signal-to-noise ratios (SNRs) were calculated using the following equations:

SNR of the left ventricle: SNR[LV] = HU[LV]/SD[air]
SNR of the right ventricle: SNR[RV] = HU[RV]/SD[air]
SNR of the ascending aorta: SNR[AAO] = HU[AAO]/SD[air]
SNR of the main PA: SNR[PA] = HU[PA]/SD[air]
SNR of the myocardium: SNR[myocardium] = HU[myocardium]/SD[air]

The contrast-to-noise ratios (CNR) were calculated using the following equations:

CNR of the left ventricle: CNR[LV] = SNR[LV] − SNR[myocardium]
CNR of the right ventricle: CNR[RV] = SNR[RV] − SNR[myocardium]
CNR of the ascending aorta: CNR[AAO] = SNR[AAO] − SNR[myocardium]
CNR of the main PA: CNR[PA] = SNR[PA] − SNR[myocardium]

### 2.4. Assessment of Subjective Image Quality

A radiologist and a pediatric cardiologist—both with more than 10 years of experience in imaging of acquired and congenital heart disease—independently assessed the image quality using a 4-point Likert-scale (1 = poor, 2 = fair, 3 = good, 4 = excellent) [37] by scoring the following nine parameters: (1) overall image noise, (2) motion artifacts, (3) high attenuation CM artifacts, (4) depiction of the aorta, (5) depiction of pulmonary arteries (i.e., truncus pulmonalis, right and left pulmonary artery), (6) depiction of the cardiac cavities (i.e., ventricles and atria), (7) depiction of the atrial and ventricular septum, (8) depiction of the veno-atrial connections and (9) overall quality of the scan. While parameters 1–3 reflect technical artifacts that influence image quality, parameters 4–9 focus on the visualization of anatomical structures which are crucial for an accurate diagnosis during a clinical reading process.

### 2.5. Analysis of Radiation Exposure

We evaluated the dose length product (DLP) (mGy × cm) for the CTA acquisitions and the bolus tracking (if applied) and calculated the effective doses (E) (mSv) thereof by the formula

E = DLP × Kappa

using the age and kV specific conversion factors (Kappa) previously published by Deak et al. [38] according to the International Commission on Radiological Protection (ICRP) publication 103 recommendation.

### 2.6. Statistical Analysis

Values for patients’ specific parameters, attenuation values, SNR and CNR values and the radiation exposure parameters (DLP and E) are given as means ± standard deviation. Analysis for normality was performed using Kolmogorov–Smirnov-tests. Depending on the normal distribution, unpaired *t*-test and Mann–Whitney tests were performed where appropriate for analysis of statistically significant differences of the means of consecutive parameters between both injection approaches. A *p*-value < 0.05 was considered statistically significant.

Ordinal values of the image quality scoring are given as medians with 95% confidence intervals. Significant differences between the quality ratings were analyzed with Wilcoxon Signed Rank test, again with a *p*-value < 0.05 considered statistically significant. Inter-observer reliability was analyzed by rank correlation with calculation of the Kendall’s Tau-b correlation coefficient (*τ*). Analogous to Abadi et al., Tau-b values were interpreted as follows: *τ* < 0, lack of agreement; *τ* ≥ 0 to ≤ 0.200, poor; *τ* > 0.200 to ≤ 0.400, fair; *τ* > 0.400 to ≤ 0.600, moderate; *τ* > 0.600 to ≤ 0.800, good; and *τ* > 0.800 to ≤ 1, strong agreement [39].

IBM^©^ SPSS 28.0 statistics software, GraphPad Software Prism 8 (Version 8.4.2) and Microsoft^©^ Excel 2016 were used for statistical analyses and handling of the data.

## 3. Results

### 3.1. Patients’ Characteristics

A total number of 137 CTA examinations performed in 120 consecutive children were included in this study. Thereof, 71 CTA studies (51.8%) were performed in male patients. Patients’ age at the date of the CT examination was median 0.5 years (mean 3.9 years, range 1 day–16.4 years). The CTAs were based on the following diagnoses: complex congenital heart malformations (76 cases), (suspected) malformations of thoracic vessels or the coronary arteries (45 cases) and acquired cardiovascular diseases (16 cases).

The number of CTAs exceeded the number of patients, as follow-up CTAs were performed on individual patients.

### 3.2. CM Administration

#### 3.2.1. Application Route of the CM

CM injection was performed either via peripheral venous lines in the upper or lower extremities or via jugular, femoral or umbilical central venous catheters. Central venous application was performed in 39/137 (28.5%) patients. Central venous lines had a minimum size of 3 French (=1 mm). Peripheral CM administration was performed in 98/137 (71.5%) CTAs. Thereof, 24-gauge (G) peripheral venous cannulas were used in 36/137 (26.3%) CTAs and 22 or 20 G in 62/137 (45.2%) CTAs.

#### 3.2.2. CM Volumes, Flow Rates, Modalities of CM Administration and Radiation Exposure

Overall mean CM volume was 17 mL ± 16 mL. In 86.9% of the cases (119/137), automated PI with bolus tracking was performed. In these cases, the mean flow rate was 1.53 ± 0.92 mL/s (range: 0.5–5.0 mL/s). Up to the age of 1 year, flow rate was 0.5 to 1.5 mL/s, and in children from 1 to 6 years of age, it was 1.0 to 2.0 mL/s. Flow rates of more than 2.0 mL/s were only used in children older than 6 years of age.

In 13.1% of the cases (18/137), contrast media and following saline bolus were applied by MI.

Automated radiation exposure control resulted in tube voltage settings of 70 kV in 118 CTAs and 80 kV in 17 CTAs; 90 kV was only applied in two patients, both of whom were 14 years old and had a body weight of 45 kg.

Table 1 shows a comparison of the patients’ characteristics and radiation exposure of all patients comparing automated PI with MI.

Radiation exposure parameters DLP and E were significantly higher in the overall PI group than in the MI group. In the PI group, the proportion of radiation exposure based on bolus tracking was 0.07 ± 0.08 mSv.

The overall results show that CTA with PI was performed in significantly older and larger patients. As one of the main contributors to radiation dose in CT is scan length, we decided to perform a subgroup analysis in order to adjust for these differences of the study populations. To create stratified samplings, we selected a number of 18 age-, height- and weight-matched individuals from the patient group receiving CM by PI and bolus tracking. Table 2 shows the comparison between the matched subgroup of these 18 patients with PI with all 18 patients with MI, indicating that no significant differences consisted between both groups regarding all parameters. Here, the fraction of radiation exposure based on bolus tracking was 0.06 ± 0.06 mSv.

#### 3.2.3. CM-Associated Complications

Application of contrast media and saline chaser bolus was uneventful for both MI and automated PI. There were no cases of associated complications such as extravasation, air embolism or allergic reactions in the whole cohort of CTA studies.

### 3.3. Image Quality

#### 3.3.1. Objective Image Quality

First, the total PI patient group was compared with the MI group. All attenuation, SNR and CNR values were significantly higher for the group with automated PI compared to MI, with the only exception for the SNR obtained from the left ventricle.

Attenuation, SNR and CNR values, as specified for the different modalities of CM application, are provided in Table 3.

Second, when comparing the 18 MI patients with the 18 matched PI patients, the values for attenuation, SNR and CNR were also significantly higher in this PI subgroup, again with the only exception for SNR values of the left ventricle (see Table 4).

#### 3.3.2. Subjective Image Quality

For both PI and MI, the median score was “good” (Likert 3) by both readers in all rated parameters apart from “CM artifacts” in the MI group (median 4 for reader 1 and for reader 2). Thus, median rating for all relevant anatomic structures was acceptable for diagnostic purposes.

**Figure 2:**
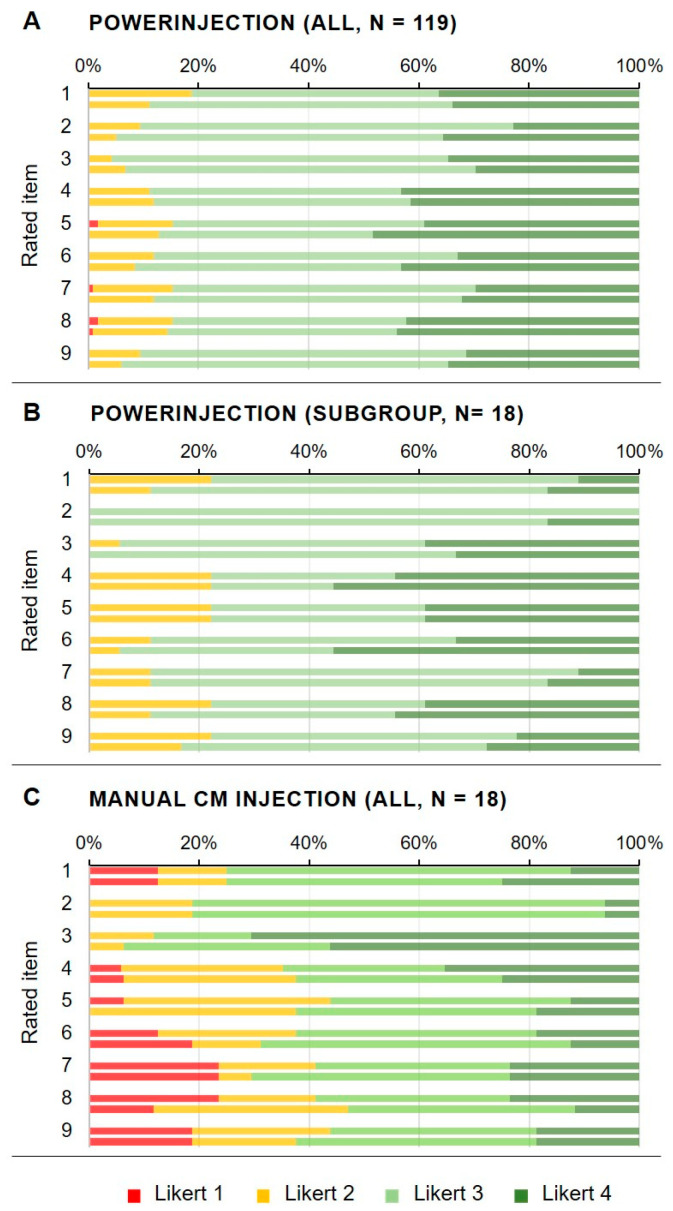
Frequency distribution of the scores of both readers based on a 4-point Likert-scale. All patients with automated power injection (PI) (**A**), the subgroup of 18 matched patients with PI (**B**) and all patients with manual injection (MI) of contrast agent (**C**). CM: contrast medium; *n*: number. The rated items (*y*-axis) are (1) overall image noise, (2) motion artifacts, (3) contrast medium artifacts, (4) depiction of aorta, (5) depiction of pulmonary arteries, (6) depiction of cardiac cavities, (7) depiction of septa, (8) depiction of venous-atrial connections and (9) overall quality of the scan. For each item, the upper bar shows the scoring of reader 1 and the lower bar the scoring of reader 2, respectively. Note that the majority of CTA studies were rated “good” or “excellent” (Likert 3 or 4, light and dark green), in particular with PI (**A**,**B**). The vast majority of inferior ratings (Likert 1 or 2, red and yellow) were addressed to CTA studies performed after MI (**C**).

Frequency distribution of scores is shown in Figure 2. Regarding the item “overall image quality”, 88.3% of cases were rated with Likert score 3 or 4 by reader 1 and in 92.0% of cases by reader 2 in the total population.

When comparing the MI group (18 CTA studies) with the matched PI subgroup (18 CTA studies), overall image quality for MI was rated inferior with Likert 3 or 4 in 61.1% for reader 1 and 66.7% for reader 2 compared to 77.7% and 83.3% in the matched PI group, respectively. The scoring “poor” (Likert 1) for “overall image quality” only occurred in the MI group.

**Figure 3:**
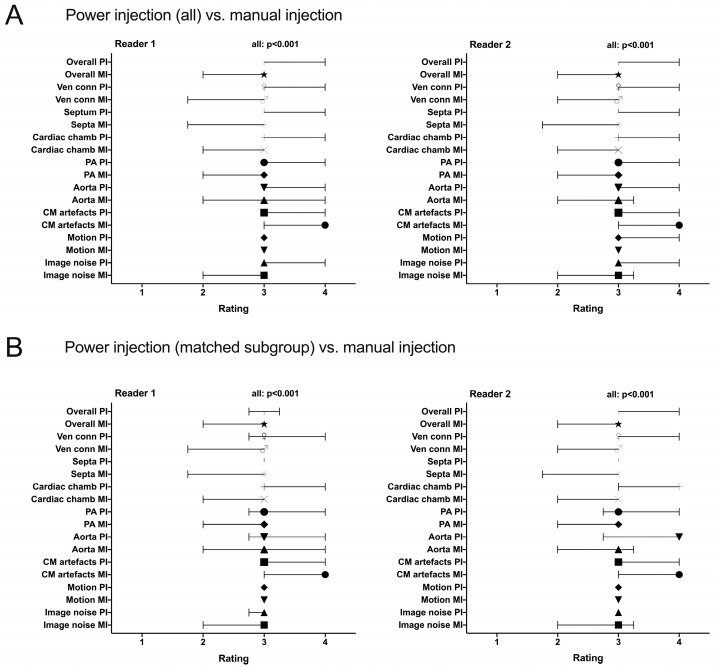
The quality ratings by reader 1 and reader 2 shown as medians and interquartile range: comparison of the total power injection group (PI) vs. the manual injection group (MI) (**A**) and comparison of the matched PI subgroup vs. the manual injection group (**B**). All items were rated significantly better for the PI group and the matched PI group compared to the MI group with the exception of “CM artifacts”. The rated items (*y*-axis) are Ven conn:depiction of the venous–atrial connections; Septa: depiction of the septa; Cardiac chamb: depiction of the cardia cavities; PA: depiction of the pulmonary arteries; Aorta: depiction of the aorta; CM: contrast medium. The different symbols mark the median of the respective rated item.

All items of the qualitative image evaluation were rated significantly better for the PI group and the matched PI group compared to the MI group with the exception of “CM artifacts”, which was rated as less apparent or influencing for CTA with MI. The interquartile ranges of scores were significantly better both in the total PI group and the matched PI group (range 3–3 to 3–4 for all items) than in the MI group (range 2–3 to 3–3) with the exception of “CM artifacts”, which was rated as less apparent or influencing for CTA with MI (3–4). See Figure 3 for this comparison.

There was a strong inter-observer reliability between reader 1 and reader 2, with a Kendall’s Tau-b correlation coefficient of *τ* = 0.802 (*p* < 0.001, 95 % confidence interval 0.788–0.815).

**Figure 4:**
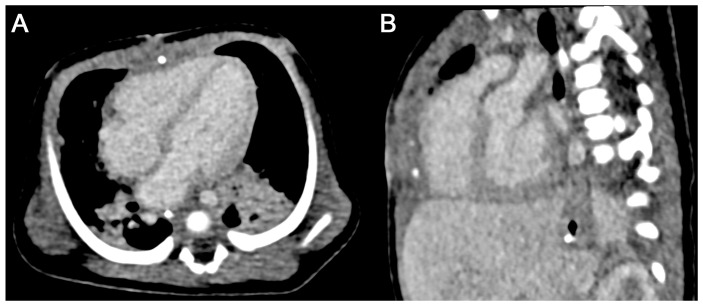
Case of a one-month-old male patient with d-transposition of the great arteries ((**A**) axial and (**B**) oblique sagittal MPR views). A total of 7 mL of contrast agent was administered by hand injection followed by 10 mL of saline. While the anatomy of the cardiac chambers and the great arteries can clearly be depicted, contrast enhancement within these structures is suboptimal. Effective radiation dose was 0.33 mSv.

**Figure 5:**
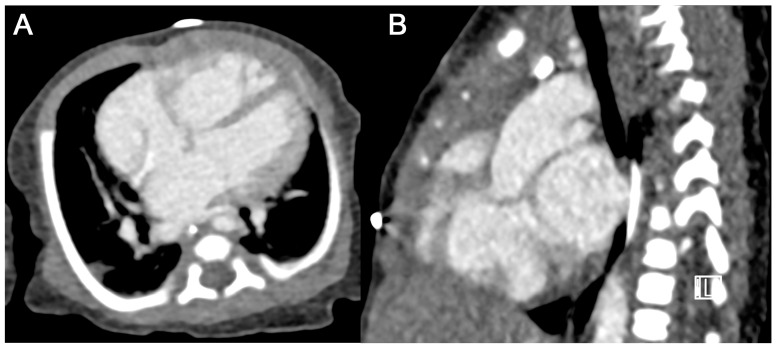
Case of a one-month-old male presenting with a truncus arteriosus communis ((**A**) axial and (**B**) parasagittal MPR views). A total of 6 mL of contrast agent was administered by hand injection followed by 10 mL of saline. Again, the anatomy of the cardiac chambers and the great arteries can be identified and delineated. Yet, contrast enhancement within the target volume might not be sufficient to identify smaller vascular structures. Effective radiation dose was 0.16 mSv.

**Figure 6:**
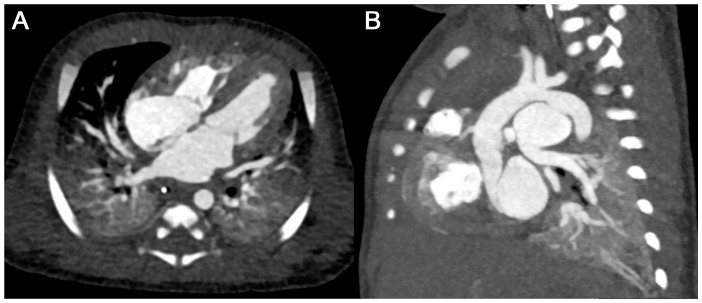
Case of a four-day-old male with aneurysm of a patent Ductus arteriosus ((**A**) axial and (**B**) parasagittal MPR views). A total of 4 mL of contrast agent was injected with a flow rate of 0.7 mL/s followed by 10 mL of saline using a power injector with bolus tracking. Note the markedly better enhancement of the cardiac chambers and great vessels as compared with Cases 1 and 2. Total effective radiation dose was 0.40 mSv (including 0.04 mSv for the bolus tracking).

**Figure 7:**
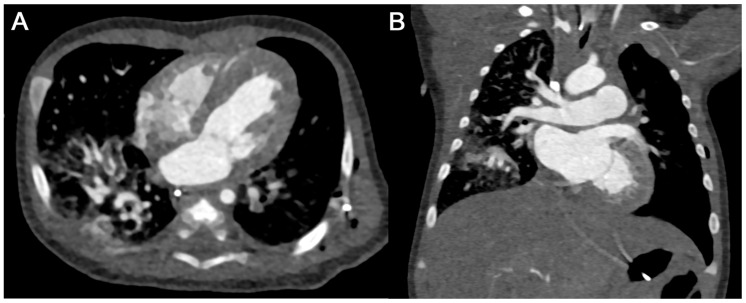
Case of six-week-old female after clipping of a patent Ductus arteriosus ((**A**) axial and (**B**) coronal MPR views). A total of 4 mL of contrast agent was injected with power injection and bolus tracking at a flow rate of 0.7 mL/s followed by 10 mL of saline, resulting in an excellent enhancement of the heart and great vessels. Total effective radiation dose was 0.42 mSv (including 0.04 mSv for the bolus tracking).

Figure 4 and Figure 5 present two patients with congenital heart disease in which CTA were performed after MI of contrast agent. Figure 6 and Figure 7 display representative cases of CTA acquired after automated PI and bolus tracking.

## 4. Discussion

The advent of modern CT technology allowing for scanning with low tube voltage settings in combination with automated tube current modulations and high-pitch acquisitions had a marked impact on the reduction in radiation exposure, in particular for imaging of the cardiovascular system [34,40].

In our study at hand, we analyzed 137 pediatric CTAs. In 119 CTAs, CM administration was performed with PI, while in 18 CTAs, MI was performed. The smallest peripheral venous cannula size was 24 gauge in 26.3% of cases with a mean CM volume of 17 mL. In PI, the mean CM flow rate was 1.52. Because patients in the PI group were significantly older and had a higher weight and height, both the entire PI group and a PI subgroup stratified by patient characteristics were compared with the MI group. The result was that the use of automated PI with bolus tracking was associated with both better objective and subjective image quality compared to CTA studies performed with MI of contrast media. When analyzing objective image quality, mean attenuation values for the anatomic structures of interest (LV, RV, aorta and pulmonary trunk) were notably high, ranging from 528 to 623 HU after PI compared to 264 to 331 HU in the MI group. The CNR values of the corresponding groups were 22.2 to 31.5 versus 10.6 to 16.0, respectively. Thus, there were both significantly higher attenuation and CNR values in the matched PI subgroup (and in the entire PI group) compared to the MI group.

In comparison, in a previous publication comparing different contrast agents with different iodine concentration for CTA after automated PI, attenuation values of coronary arteries reached 426–466 HU [41]. Here, tube voltage was manually set to 100 kV or 120 kV, depending on whether the patients’ body weight was more or less than 80 kg. In contrast, the tube voltage in our study was automatically set by the scanner, resulting in 70–80 kV in the majority of cases. This might contribute to the even higher attenuation and CNR values obtained in our evaluation [42].

Zapala et al. compared manual and automated contrast media applications for pulmonary CTA in children, showing attenuation values of 329–340 HU for the pulmonary arteries [21], without significant differences between the two injection methods. The attenuation values reported in this paper are comparable with those of our subgroup of CTA studies acquired with MI without bolus tracking. In contrast, mean attenuation values of the pulmonary arteries in CTAs with PI and bolus tracking reached 568 HU in our study, which is markedly higher than in the work of Zapala et al. This emphasizes the role of bolus tracking, which is crucial for the timing of the CTA acquisition at the moment of high contrast media concentration in the target vessel.

Nagy et al. report on attenuation values using bolus tracking that were very similar to our findings [43]. Here, CM with 300 mg/dl iodine was applied using different injection modes. However, contrast agent volumes used in their approach were 1.7 to 2 mL per kg body weight, which is almost twice as high as the volumes used in our evaluation.

Saake et al. also compared MI and PI without the use of bolus tracking [44]. Here, PI also resulted in significantly higher values for SNR and CNR. The main difference to our study lies in the volumes and concentrations of contrast medium applied. Saake et al. propagated injection of diluted contrast agent with larger volumes, which were up to four times higher than in our population, while also applying higher mean injection rates of 1.9 mL/s. Especially in children with CHD and associated cardiac congestion, volume loading could be disadvantageous. In contrast, we used highly concentrated contrast agent without dilution applied at rather low flow rates. In combination with bolus tracking, this “low-volume, low-flow approach” resulted in comparable values of attenuation, SNR and CNR as compared with the approach of Saake et al.

In another recent publication, automated contrast injection with and without bolus tracking in children up to one year of age were compared [45]. In contrast to our study, again markedly higher volumes (4 mL/kg body weight) of diluted contrast agent were applied. No significant differences in contrast attenuation of the aorta and pulmonary artery were found between the two approaches. At the same time, the radiation exposure in the group of patients who were scanned with bolus tracking was significantly higher, with an additional dose of 0.15 mSv for bolus tracking acquisition. However, this is in contrast to our findings between the matched group of patients with PI and MI, where we found no significant differences in total radiation exposure. In our subgroup, mean dose attributable to bolus tracking was 0.06–0.07 mSv, which is less than half the radiation dose of 0.15 mSv reported for bolus tracking in the patient cohort of Yoshiura et al. Importantly, the tube voltage for bolus tracking in our study was set lower at 70 kV, which may partly explain the lower dose in our population.

Due to the rapid contrast agent distribution in small children, CTA acquisition must be acquired relatively soon after the injection. In the case of an MI approach, the examiner is usually unable to leave the scanner room in time before the start of the spiral acquisition. This results in additional radiation exposure for the examiner, which will not be the case when using PI. The latter can be performed from outside the scanner room, which is another advantage of this technique.

As described by Chatzaraki et al., there are different formulas applied for SNR and CNR calculation [36]. In our study, the standard deviation of air attenuation was used as image noise. Comparable to other studies evaluating thoracic CTA [36,43] or coronary CTA [46], we achieved satisfactorily high CNR values of above 20 in all relevant structures using PI with bolus tracking, while they were clearly below 20 in the MI group.

In our evaluation, the subjective image quality was assessed by two independent readers applying a 4-point Likert scale. The median rating of the displayed relevant cardiac structures was “good”, featuring a strong inter-observer reliability. Thus, anatomy and underlying malformations could be reliably diagnosed [37]. Here, too, the objective quality in the PI group was significantly better than in the MI group. Only CM artifacts occurred less frequently in the MI patients, which might be attributed to the lower CM flow rates in the case of hand injection.

In our patient cohort, CM was mostly (71.5% of cases) administered via peripheral venous cannulas of minimal 24 G. Regardless of the venous access, CM injection was performed automatically with power injectors in the vast majority of cases. The flow rates of 0.5–5.0 applied in our patients were similar to those reported by Dien Esquivel et al. (here, 1.0–4.5 mL/s in children and adolescents) and by Xie et al. (here, 0.5–2.0 mL/s in children up to 6 years of age) [47,48]. However, as part of our clinical routine, venous access devices were always inserted and tested with caution by performing test injection with sterile saline by hand and with the power injector at the targeted flow rate. If the proper function of the venous access is questionable, we would not recommend injecting CM through it, neither with MI nor with automated PI [49]. Our approach contrasts with previous studies that favored manual injection for 24- and 22-gauge cannulas depending on the anatomical application site. [21,24]. However, there were no CM-associated complications in our patients. In the literature, incidence of peripheral CM extravasation when using power injectors accounts for 0.1–0.9% [22,24,47]. Another possible complication that has to be considered is air embolism, especially in patients with cardiovascular malformations and right-to-left shunt resulting in possible cerebral affection [24]. Care must therefore be taken to ensure that the lines and injection pistons are carefully vented before they are connected to the patients.

For the assessment of the relevant radiation dose, the effective dose E as the sum of the tissue-equivalent doses of organs and tissues is the decisive measure. Tissue- and age-specific weighting factors are provided by the International Commission on Radiological Protection (ICRP) [38,50]. Goodman et al. describe the reduction in radiation exposure to children by cranial and abdominal CT within the last 20 years from >50 mSv to recently about 1 mSv or less [31]. Third-generation DSCT scanners allow high peak tube current with low kilovoltage and prospectively ECG-triggered high-pitch spiral acquisition, thereby generating lower radiation exposure [40].

The radiation dose to be achieved must be as low as possible, especially for pediatric patients, as children are more sensitive to ionizing radiation than adults. Causes for the higher vulnerability are a longer life expectancy and a higher cellular proliferation rate in growing children [31,51]. An exact threshold of radiation dose for stochastic effects does not exist, although the linear no-threshold model is controversially discussed [52], and other studies query the evidence of carcinogenicity of low radiation doses [53]. On the other hand, there is a higher risk for malignant diseases caused by high radiation doses. This does not only apply to survivors of the atomic bomb [54]. A large Australian study revealed an increased incidence for cancer following CT scans in childhood [55]. Pearce et al. showed that a cumulative ionizing radiation dose by CT of about 60 mGy triples the risk for brain tumors and a dose of about 50 mGy triples the risk for leukemia [29]. Yet, the cumulative doses described here appear to be very high and are significantly higher than the doses we currently monitor with modern CT scanners. In our study, we achieved a mean effective dose below 1 mSv for both injection approaches, MI and PI with bolus tracking. This would correspond to about 50 to 100 CT examinations to achieve a cumulative radiation dose of 50 mGy or more, as mentioned in the earlier studies. Bolus tracking increased the radiation doses by 0.07 mSv in the overall PI group, which appears acceptable, as this approach results in high image quality and contributes to an accurate diagnosis.

Compared to our study, former publications including fewer patients showed similar radiation doses for CTA in children with congenital cardiac malformations: Tada et al. [56], Al-Mousily et al. [57] and Zhang et al. [58] described 1.24 ± 0.42 mSv, 0.8 ± 0.39 mSv and 0.42 ± 0.08 mSv, respectively. It is noteworthy that these doses are all lower than the global average effective dose rate of the natural background from terrestrial and cosmic radiation, which amounts to about 2.4 mSv per year [59].

In cardiovascular malformations, exact anatomical evaluation is indispensable for planning of interventional and surgical procedures and for follow-up. For CTA, several studies provided excellent results in diagnostic accuracy of congenital heart defects [60] and thoracic vessels [61,62,63] which are consistent with the presented results. CTA is suitable primarily for the following purposes: diagnosis of aortic malformations and outflow tract malformations; evaluation of the pulmonary arteries in complex heart defects; the planning of arterial switch operation; planning of the correction of anomalous pulmonary venous connections; and assessment of compression of adjacent organs by thoracic cardiovascular malformations (i.e., “vascular rings and slings”). Three-dimensional reconstruction features further information for subsequent surgical or interventional procedures considering the vascular and mediastinal anatomy [15,64]. Moreover, CTA is an appropriate non-invasive method for the evaluation of anomalous origin, structure or course of coronary arteries [40,56].

### Limitations

Limitations of our study are the retrospectivity and single-center design with a limited number of included patients. The inclusion of several different imaging centers would have increased the number of CTA examinations and possibly led to a more homogeneous patient cohort. In addition, the uneven distribution of injection modes reflects to some extent the evolution of CT scans in pediatric patients over the given time period and the gain in personal expertise at our center, which might be considered as a certain bias. Furthermore, our evaluation was focused on children with mainly congenital heart disease representing a selection bias. However, we are convinced that other clinical indications for thoracic CT studies would benefit in the same way of better image quality achieved by contrast application with PI. Moreover, including individuals with different ages up to 18 years and providing a rather heterogeneous population may explain the scattered values of attenuation, SNR and CNR.

Furthermore, it remains unclear whether the improved image quality of CTA performed with PI will have a direct impact on the treatment of children with congenital heart defects. This will be difficult to assess objectively. One possible measure, although this is only based on personal experience at our site, is the fact that our cooperating pediatric cardiologists and cardiac surgeons have increasingly requested CTA examinations instead of invasive angiographies or magnetic resonance angiographies in recent years. This cannot be scientifically proven within this study but could be the topic of a further evaluation.

## 5. Conclusions

State-of-the-art CTA contributes important diagnostic information to the treatment of cardiac diseases in children of all ages, providing good image quality even though low CM volumes are administered with low flow rates via small venous access devices. In particular, modern techniques such as automated radiation exposure and use of automated contrast agent PI in combination with bolus tracking contribute to a consistently high and reproducible image quality. It appears to be superior to manual contrast injection regarding both objective and subjective image quality.

Thoracic CTA can be acquired in children routinely with consistently low radiation exposure below 1 mSv in all pediatric age groups. It represents a safe and fast non-invasive imaging approach in the work-up of various congenital and acquired cardiovascular diseases.

## Figures and Tables

**Table 1:** Patients’ characteristics and radiation exposure of all patients with PI versus all patients with MI. Note that the patients receiving PI were significantly older, taller and heavier. Based on the automated radiation exposure control applied for the CTA acquisition, this results in higher radiation exposure values (CTDI_vol_, DLP and E) for this subgroup.

Parameter	PI (All) *n* = 119	MI (All) *n* = 18	*p*
Male/female, *n*	58/61	13/5	
Age (years)	4.4 ± 5.3	0.59 ± 0.95	0.04
Body weight (kg)	17.1 ± 16.2	6.1 ± 2.7	0.05
Height (m)	0.95 ± 0.41	0.63 ± 0.12	0.03
BMI (kg/m^2^)	15.1 ± 3.2	14.8 ± 2.0	0.74
CTDI_vol_ (mGy)	1.13 ± 1.51	0.45 ± 0.22	0.01
DLP (mGy × cm)	23.4 ± 26.2	7.8 ± 4.6	0.003
E (mSv)	0.83 ± 0.7	0.53 ± 0.3	0.04

Data are expressed as means ± SD. PI: automated power injection with bolus tracking; MI: manual injection; *n*: number; BMI: body mass index; CTDI_vol_: volume computed tomography dose index; DLP: dose length product; E: effective dose.

**Table 2:** Patients’ characteristics and radiation exposure parameters of the matched subgroup of patients with PI versus all patients with MI. There were no significant differences in the patient metrics between the two injection groups. Accordingly, the radiation exposure parameters (CTDI_vol_, DLP and E) were also not significantly different.

Parameter	PI (Matched Subgroup) *n* = 18	MI (All) *n* = 18	*p*
Male/female, *n*	11/7	13/5	
Age (years)	0.71 ± 1.03	0.59 ± 0.95	0.57
Body weight (kg)	6.6 ± 3.2	6.1 ± 2.7	0.74
Height (m)	0.65 ± 1.3	0.63 ± 0.12	0.64
BMI (kg/m^2^)	14.6 ± 1.7	14.8 ± 2.0	0.90
CTDI_vol_ (mGy)	0.44 ± 0.2	0.45 ± 0.22	0.76
DLP (mGy × cm)	6.8 ± 3.5	7.8 ± 4.6	0.62
E (mSv)	0.52 ± 0.3	0.53 ± 0.3	0.76

Data are expressed as means ± SD. PI: automated power injection with bolus tracking; MI: manual injection; *n*: number; BMI: body mass index; CTDI_vol_: volume computed tomography dose index; DLP: dose length product; E: effective dose.

**Table 3:** Measured attenuation values, calculated SNR and CNR of the target structures (all patients with PI versus all patients with MI).

Structure	Attenuation (HU)			SNR			CNR		
	PI (All) *n* = 119	MI (All) *n* = 18	*p*	PI (All) *n* = 119	MI (All) *n* = 18	*p*	PI (All) *n* = 119	MI (All) *n* = 18	*p*
LV	547 ± 218	331 ± 184	<0.001	29.6 ± 13.1	25.9 ± 20.5	0.11	22.2 ± 11.3	16.0 ± 17.2	0.007
RV	569 ± 279	264 ± 132	<0.001	30.5 ± 15.6	19.9 ± 11.2	<0.001	22.9 ± 14.9	10.6 ± 8.7	<0.001
AAO	577 ± 228	325 ± 171	<0.001	34.6 ± 14.2	25.6 ± 23.7	<0.001	27.1 ± 13.2	15.7 ± 20.4	<0.001
PA	568 ± 298	294 ± 141	<0.001	34.4 ± 19.4	21.5 ± 12.6	<0.001	27.4 ± 18.5	11.6 ± 10.1	<0.001

Data are expressed as means ± SD. HU: Hounsfield units; SNR: signal-to-noise ratio; CNR: contrast-to-noise ratio; PI: automated power injection with bolus tracking; MI: manual injection; *n*: number; LV: left ventricle; RV: right ventricle; AAO: ascending aorta; PA: main pulmonary artery.

**Table 4:** Measured attenuation values, calculated SNR and calculated CNR of the target structures of the matched subgroup of patients with PI versus all patients with MI.

Structure	Attenuation (HU)			SNR			CNR		
	PI (Subgroup) *n* = 18	MI (All) *n* = 18	*p*	PI (Subgroup) *n* = 18	MI (All) *n* = 18	*p*	PI (Subgroup) *n* = 18	MI (All) *n* = 18	*p*
LV	621 ± 228	331 ± 184	<0.001	35.3 ± 17.1	25.9 ± 20.5	0.07	27.2 ± 14.6	16.0 ± 17.2	0.009
RV	528 ± 262	264 ± 132	<0.001	30.8 ± 16.3	19.9 ± 11.2	0.008	22.7 ± 15.0	10.6 ± 8.7	0.007
AAO	660 ± 285	325 ± 171	<0.001	39.6 ± 19.0	25.6 ± 23.7	0.005	31.5 ± 16.7	15.7 ± 20.4	0.001
PA	521 ± 259	294 ± 141	0.002	32.7 ± 15.1	21.5 ± 12.6	0.02	24.6 ± 13.9	11.6 ± 10.1	0.003

Data are expressed as means ± SD. HU: Hounsfield units; SNR: signal-to-noise ratio; CNR: contrast-to-noise ratio; PI: automated power injection with bolus tracking; MI: manual injection; *n*: number; LV: left ventricle; RV: right ventricle; AAO: ascending aorta; PA: main pulmonary artery.

## Data Availability

The data underlying the present study are available on reasonable request (corresponding author).

## Abbreviations

The following abbreviations are used in this manuscript:

AAO	ascending aorta
CM	contrast medium
CNR	contrast-to-noise ratio
CT	computed tomography
CTA	computed tomography angiography
CTDI_vol_	volume computed tomography dose index
DLP	dose length product [mGy × cm]
DSCT	dual-source computed tomography
E	effective dose [mSv]
G	Gauge
HU	Hounsfield unit(s)
IVS	interventricular septum
LV	left ventricle
LVPW	left ventricular posterior wall
MI	manual CM injection
PA	main pulmonary artery
PI	automated power injection with bolus tracking
ROI	region of interest
RV	right ventricle
SD	standard deviation
SNR	signal-to-noise ratio
